# Mucosal antibody responses to SARS-CoV-2 booster vaccination and breakthrough infection

**DOI:** 10.1128/mbio.02280-23

**Published:** 2023-12-01

**Authors:** Disha Bhavsar, Gagandeep Singh, Kaori Sano, Charles Gleason, Komal Srivastava, PARIS Study Group, Annika Oostenink, Juan Manuel Carreno, Viviana Simon, Florian Krammer

**Affiliations:** 1 Department of Microbiology, Icahn School of Medicine at Mount Sinai, New York, New York, USA; 2 Center for Vaccine Research and Pandemic Preparedness (C-VaRPP), Icahn School of Medicine at Mount Sinai, New York, New York, USA; 3 Department of Pathology, Molecular and Cell-Based Medicine, Icahn School of Medicine at Mount Sinai, New York, New York, USA; 4 Division of Infectious Diseases, Department of Medicine, Icahn School of Medicine at Mount Sinai, New York, New York, USA; 5 The Global Health and Emerging Pathogens Institute, Icahn School of Medicine at Mount Sinai, New York, New York, USA; St Jude Children's Research Hospital, Memphis, Tennessee, USA

**Keywords:** sIgA, SARS-CoV-2, saliva, spike, breakthrough infection, booster vaccination, mRNA vaccine

## Abstract

Coronavirus disease 2019 (COVID-19) vaccines have saved millions of lives. However, variants of severe acute respiratory syndrome coronavirus 2 (SARS-CoV-2) have emerged causing large numbers of breakthrough infections. These developments necessitated the rollout of COVID-19 vaccine booster doses. It has been reported that mucosal antibody levels in the upper respiratory tract, especially for secretory IgA (sIgA), correlate with protection from infection with SARS-CoV-2. However, it is still unclear how high levels of mucosal antibodies can be induced. In this study, we measured serum IgG, saliva IgG, and saliva sIgA responses in individuals who received COVID-19 mRNA booster vaccinations or who experienced breakthrough infections. We found that mRNA booster doses could induce robust serum and saliva IgG responses, especially in individuals who had not experienced infections before, but saliva sIgA responses were weak. In contrast, breakthrough infections in individuals who had received the primary mRNA vaccination series induced robust serum and saliva IgG as well as saliva sIgA responses. Individuals who had received a booster dose and then had a breakthrough infection showed low IgG induction in serum and saliva but still responded with robust saliva sIgA induction. These data suggest that upper respiratory tract exposure to antigen is an efficient way of inducing mucosal sIgA while exposure via intramuscular injection is not.

**IMPORTANCE:**

Antibodies on mucosal surfaces of the upper respiratory tract have been shown to be important for protection from infection with SARS-CoV-2. Here we investigate the induction of serum IgG, saliva IgG, and saliva sIgA after COVID-19 mRNA booster vaccination or breakthrough infections.

## OBSERVATION

Vaccines for coronavirus disease 2019 (COVID-19) have saved millions of lives (1). However, variants of severe acute respiratory syndrome coronavirus 2 (SARS-CoV-2) that escape neutralizing antibody responses have significantly decreased vaccine effectiveness against both infection and symptomatic disease (2). Importantly, in order to protect from infection, robust titers of neutralizing mucosal antibodies in the upper respiratory tract are likely needed. In fact, mucosal antibody titers have been linked to protection from infection (3–5). However, injected vaccines are not very effective in inducing mucosal immunity in the upper respiratory tract (6). Right after vaccination, when IgG serum titers are very high, IgG titers sufficient for protection are likely present on mucosal surfaces of the upper respiratory tract too. However, as titers wane, these IgG levels likely decline to sub-protective levels. The initial efficacy of protection from infection of the mRNA vaccines which declined quickly supports this hypothesis (7). However, secretory IgA (sIgA), which is actively transported to mucosal surfaces and is produced by B cells in the lamina propria below these surfaces is typically not induced by vaccination. It is believed that these mucosa-specific sIgA responses are mostly induced when antigen is encountered via the mucosal route, e.g., after infection with a respiratory virus or potentially after mucosal vaccination—but not during vaccination with injected vaccines. We have in the past shown that mRNA vaccination in previously naïve individuals results in mucosal IgG but not mucosal sIgA responses while sIgA was induced by mRNA vaccination in individuals who already had been infected before vaccination with SARS-CoV-2 (6). Here, we explored the induction of mucosal IgG and sIgA responses in individuals who received booster vaccine doses (third dose) or who had breakthrough infections after the primary vaccination series in our Protection Associated with Rapid Immunity to SARS-CoV-2 (PARIS) cohort (8).

### Mucosal antibody responses after mRNA booster dose

**FIG 1:**
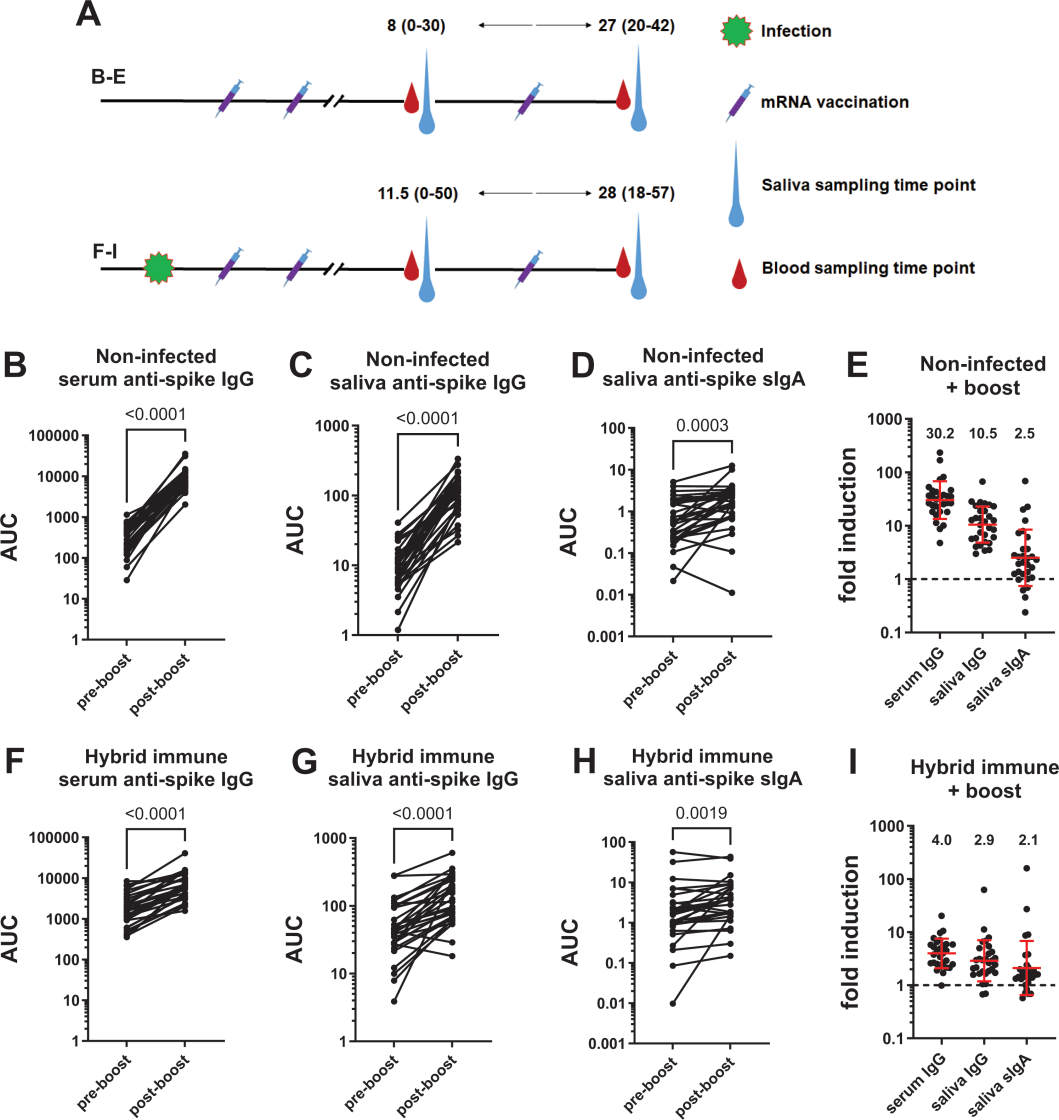
Induction of anti-spike serum IgG, saliva IgG, and saliva sIgA after COVID-19 mRNA booster vaccination. Overview of samples (**A**). Pre- and post-boost serum IgG (**B**), saliva IgG (**C**), and saliva sIgA (**D**) titers in non-infected individuals who received the primary vaccination series. (**E**) shows fold induction of absolute titers presented in panels A, B, and C. Pre- and post-boost serum IgG (**F**), saliva IgG (**G**), and saliva sIgA (**H**) titers in hybrid immune individuals who received the primary vaccination series. (**I**) shows fold induction of absolute titers presented in panels A, B, and C. AUC = area under the curve. Statistical analysis in panels A, B, C, E, F, and G was performed using a ratio-paired *t* test. The red bar in panels D and H indicates the geometric mean, and the error bars indicate the standard deviation of the geometric mean. The dotted lines indicate no induction (1-fold). *N* = 29–30 for each panel. A “2P” version of the ancestral spike was used for the serum IgG measurements, and the HexaPro version was used for the saliva IgG and sIgA measurements.

Our cohort included individuals who were naïve before they received the primary COVID-19 mRNA vaccination series (*n* = 30, 18 females) and individuals who were infected and then received the primary COVID-19 mRNA vaccination series (hybrid immune; *n* = 30, 19 females). We wanted to see what happened to serum and mucosal antibody titers when these individuals received the booster dose (third dose) of mRNA vaccine. For previously naïve individuals pre-boost samples were taken at a median of 8 days before vaccination (range 0–30 days) and post-boost samples were taken at a median of 27 days after vaccination (range 20–42 days) (Fig. 1A). For hybrid immune individuals samples were taken at a median of 11.5 days (range 0–50 days) before the booster dose and at a median of 28 days (range 18–57 days) after the booster dose (Fig. 1A). Individuals without prior infection responded with a robust serum IgG response (Fig. 1B and E, 30.2-fold induction) and saliva IgG titer increased as well (Fig. 1C and E, 10.5-fold). While mucosal sIgA was also significantly induced (Fig. 1D and E), this induction was much less robust and only 2.5-fold. For hybrid immune individuals, we found a lower level of induction of IgG in serum (Fig. 1F and I, 4.0-fold) and saliva (Fig. 1G and H, 2.9-fold), likely due to higher pre-boost titers. Saliva sIgA was induced but only 2.1-fold (Fig. 1H and I).

### Mucosal antibody responses after SARS-CoV-2 breakthrough infections

**FIG 2:**
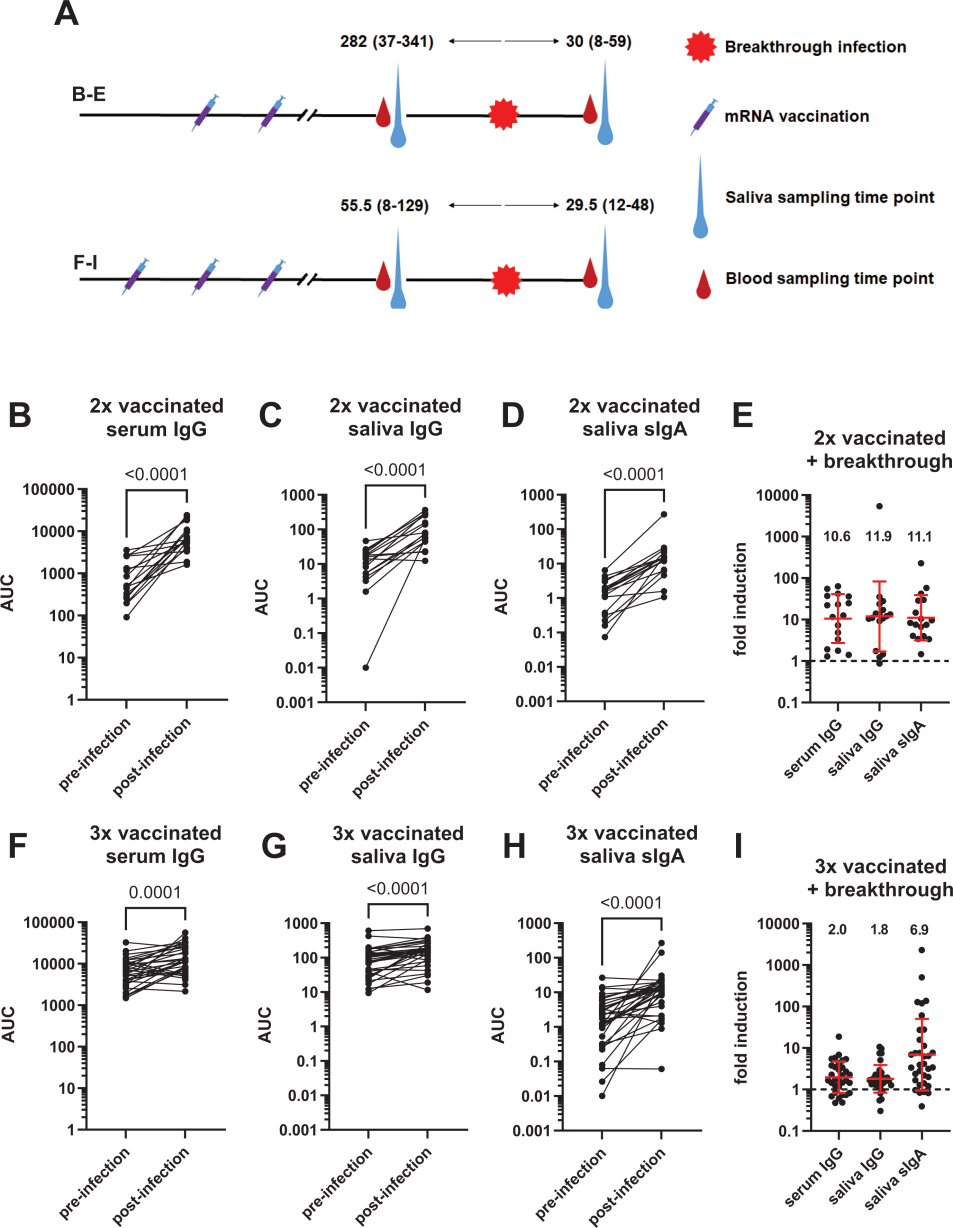
Induction of anti-spike serum IgG, saliva IgG, and saliva sIgA after SARS-CoV-2 breakthrough infections. Overview of samples (**A**). Pre- and post-breakthrough serum IgG (**B**), saliva IgG (**C**), and saliva sIgA (**D**) titers in individuals who received the primary vaccination series. (**E**) shows fold induction of absolute titers presented in panels A, B, and C. Pre- and post-breakthrough serum IgG (**F**), saliva IgG (**G**), and saliva sIgA (**H**) titers in individuals who had their breakthrough infections after the booster dose. (**I**) shows fold induction of absolute titers presented in panels A, B, and C. AUC = area under the curve. Statistical analysis in panels A, B, C, E, F, and G was performed using a ratio-paired *t* test. The red bar in panels D and H indicates the geometric mean, and the error bars indicate the standard deviation of the geometric mean. The dotted lines indicate no induction (1-fold). *N* = 17 for panels A, B, C, and D, and *N* = 32–34 for the remaining panels. A “2P” version of the ancestral spike was used for the serum IgG measurements, and the HexaPro version was used for the saliva IgG and sIgA measurements.

As a next step, we wanted to evaluate the serum IgG, saliva IgG, and saliva sIgA response after breakthrough infections. Here, we split out participants into individuals who had their breakthrough infections after the primary mRNA COVID-19 vaccination series (*n* = 17, 13 females, 3 Delta breakthroughs, 14 Omicron breakthroughs) or after the mRNA COVID-19 booster dose (*n* = 34, 26 females, all Omicron breakthroughs). For individuals vaccinated only with the primary vaccination series pre-breakthrough samples were taken at a median of 282 days before vaccination (range 37–341 days) and post-breakthrough samples were taken at a median of 30 days after vaccination (range 8–59 days) (Fig. 2A). For boosted individuals samples were taken at a median of 55.5 days (range 8–129 days) before the booster dose and a medianof 29.5 days (range 12–48 days) after the booster dose (Fig. 2A). For breakthrough infections after the primary vaccination series we found robust induction of serum IgG, saliva IgG, and saliva sIgA (Fig. 2B through E) with 10.6-fold, 11.9-fold, and 11.1-fold induction, respectively. Antibody induction in breakthrough cases after the booster dose was detectible for serum IgG and saliva IgG but less pronounced with 2.0-fold and 1.8-fold inductions, respectively (Fig. 2F, G, and I). However, the sIgA induction was still robust (6.9-fold) but was very heterogeneous (Fig. 2H and I). Of note, a possible caveat here is, that the time from sampling to breakthrough infection was much longer in individuals who had been vaccinated twice compared to individuals who were vaccinated three times and this could of course bias our observations.

### Conclusions

In summary, we observed that booster vaccination in previously naïve individuals induced strong serum IgG responses and saliva IgG responses but the induction of saliva sIgA was low. In individuals with hybrid immunity, antibody induction after the booster dose was lower in general, likely owing to higher baseline titers. Breakthrough infections after the primary vaccination series resulted in robust induction of serum IgG, saliva IgG, and saliva sIgA. However, breakthrough infection after the booster dose led to a lower level of IgG induction in serum and saliva while sIgA induction in saliva was still robust, even though there was a lot of variation among individuals. Our data suggest that breakthrough infections induce robust mucosal sIgA while injected booster doses of COVID-19 mRNA vaccines do not. This is in line with the concept that mucosal antigen delivery is needed for efficient induction of sIgA in the upper respiratory tract. However, if mucosal vaccination will induce similar levels of sIgA in the upper respiratory tract and if this leads to improved protection will need to be established in clinical trials.

## Data Availability

All data are available from ImmPort under the following identifier: SDY2398.

## SUPPLEMENTAL MATERIAL

Supplemental Material mbio.02280-23-s0001.pdf Detailed methods.

ASM does not own the copyrights to Supplemental Material that may be linked to, or accessed through, an article. The authors have granted ASM a non-exclusive, world-wide license to publish the Supplemental Material files. Please contact the corresponding author directly for reuse.
