# Supplementary material for: Mucosal antibody responses to SARS-CoV-2 booster vaccination and breakthrough infection
Source: mBio. 2023 Dec 1;14(6):e02280-23. doi: 10.1128/mbio.02280-23 (PMC10790573; doi:10.1128/mbio.02280-23)
Supplement: Supplemental Material — Detailed methods. [file mbio.02280-23-s0001.pdf]

Supplemental Material for:

Mucosal antibody responses to SARS-CoV-2 booster vaccination and breakthrough infection

Disha Bhavsar[1,2,$], Gagandeep Singh[1,2,$], Kaori Sano[1,2,$], Charles Gleason[1,2], Komal Srivastava[1,2], PARIS Study Group[1,2], Juan Manuel Carreño[1,2], Viviana Simon[1,2,3,4,5] and Florian Krammer[1,2,3]*

[1]*Department of Microbiology, Icahn School of Medicine at Mount Sinai, New York, NY, USA*

[2]*Center for Vaccine Research and Pandemic Preparedness (C-VaRPP), Icahn School of Medicine at Mount Sinai, New York, NY, USA*

[3]*Department of Pathology, Molecular and Cell-Based Medicine, Icahn School of Medicine at Mount Sinai, New York, NY, USA*

[4] *Division of Infectious Diseases, Department of Medicine, Icahn School of Medicine at Mount Sinai, New York, NY, USA*

[5]*The Global Health and Emerging Pathogens Institute, Icahn School of Medicine at Mount Sinai, New York, NY, USA*

[$]*Contributed equally*

*To whom correspondence should be addressed: florian.krammer@mssm.edu

[#]PARIS Study Group (alphabetical order): Dalles Andre, Maria C. Bermúdez-González, Dominika Bielak, Gianna Y. Cai, Christian Cognigni, Yuexing Chen, Miriam Fried, Hyun Min Kang, Giulio Kleiner, Neko Lyttle, Jacob Mauldin, Jacob Mischka, Brian Monahan, Sara Morris, Jessica Nardulli, Annika Oostenink, Ashley-Beathrese Salimbangon, Leeba Sullivan, Morgan Van Kesteren, Temima Yellin

Methods and materials

Human samples (participant enrollment, serum and saliva collection and methods)

Serum and saliva samples were collected from participants in the longitudinal observational PARIS (Protection Associated with Rapid Immunity to SARS-CoV-2) study (1). The study was reviewed and approved by the Mount Sinai Hospital Institutional Review Board (IRB-20-03374/Study-20-00442). All participants signed written consent forms prior to sample and data collection. All participants provided permission for sample banking and sharing.

Recombinant proteins

Recombinant SARS-CoV-2 spike (S) protein was produced using a mammalian cell protein expression system as described in detail by (2, 3). In brief, the S gene sequence of the ancestral spike of SARS-CoV-2 (GenBank: MN908947) was modified to include either two (2P) or six prolines (HexaPro (4)) and cloned into the mammalian expression vector pCAGGs. The spike was then expressed using the Expi293 Expression System (Thermo Fisher Scientific), according to the manufacturer's instructions. Cell culture supernatants were centrifuged at 4000 x g, filtered, and purified with Ni-nitriloacetic acid (NTA) agarose (Qiagen). Purified S protein was concentrated and buffer was exchanged to phosphate buffered saline (PBS) (pH7.4) using Amicon Ultracell (Merck) centrifugation units. Proteins were stored at -80°C until use.

Antigen specific IgG and sIgA ELISA

Immulon 4 HBX 96-well microtiter plates (Thermo Fisher Scientific, #3855) were coated with recombinant proteins (100 ng/well) in PBS (pH 7.4) overnight at 4°C. The '2P' version of the ancestral spike was used for serum IgG measurements, the HexaPro version of the ancestral spike was used for saliva IgG and sIgA measurements. Well contents were discarded and blocked with 200 µl of 3% non-fat milk (AmericanBio, # AB10109-01000,) in PBS containing 0.1% Tween-20 (PBST) (5% non-fat milk in PBST for saliva IgG and sIgA) for one hour at room temperature (RT). After blocking, 100 µl of serum samples (starting at 1:80 and serially diluted three-fold with 1% non-fat milk in PBST) or 50 µl of saliva samples (starting at 1:2 and serially diluted two-fold with 2.5% non-fat milk in PBST) were added to each well. Samples were incubated either at RT for two hours or overnight at 4°C for IgG or sIgA measurement respectively and plates were washed with PBST three times. For serum IgG measurement, 50 µl of horseradish peroxidase (HRP)-labeled goat anti-human IgG antibody (Sigma-Aldrich, #A0170) diluted 3000-fold with 1% non-fat milk in PBST were added to each well and incubated at RT for 1 hour. For saliva IgG measurement, 50 µl of HRP-labeled goat anti-human IgG antibody (Invitrogen, #31412) diluted to 0.32 µg/ml with 2.5% non-fat milk in PBST were added to each well and incubated at RT for 1 hour. For sIgA measurement, 50 µl of mouse anti-human secretory IgA antibody (MilliporeSigma, #HP6141) diluted to 5 µg/ml with 2.5% non-fat milk in PBST was added to each well and incubated at RT for 2 hours. These plates were washed again with PBST three times, and 50 µl of HRP labeled goat anti-mouse IgG Fc antibody (Invitrogen, #31439) diluted to 1:1000 with 2.5% non-fat milk in PBST was added to each well and incubated at RT for 1 hour. For all measurements, after the final washing step (three times with PBST), 100 µl of SIGMAFAST o-phenylenediamine dihydrochloride substrate solution (Sigma-Aldrich, #P9187) were added to each well at RT for 10 minutes. The reaction was stopped by addition of 50 µl of 3M hydrochloric acid (HCl). Optical density at 490 nm was measured using a Synergy 4 (BioTek) plate reader. Eight wells on each plate received no primary antibody (blank wells) and the optical density in those wells was used to assess background. Area under the curve (AUC) was calculated by deducting the average of blank values plus three times the standard deviation of the blank values. Antigen specific sIgA AUC values were adjusted by dividing the values by total IgA concentration within saliva samples (adjusted AUC). Of note, different secondary HRP-labeled anti-IgG antibodies were used because protocols were originally developed independently by different individuals in different laboratories.

Measurement of total IgA concentration within saliva samples

Immulon 4 HBX 96-well microtiter plates were coated with 250 ng/well of goat anti-human IgA (Bethyl Laboratories, #A80-102A) in PBS (pH 7.4) overnight at 4°C. Well contents were discarded and 200 µL/well of 5% non-fat milk in PBST were added and plates were incubated for one hour at RT. After blocking, 50 µL of saliva samples diluted (starting at 1:256 and serially diluted three-fold) in 2.5% non-fat milk PBST were added to each well and incubated at RT for two hours. Plates were washed with PBST three times and 50 µl/well of HRP-labeled goat anti-human IgA antibody (Bethyl Laboratories, #A80-102P) diluted to 1:10,000 in 2.5% non-fat milk in PBST was added to each well and incubated at RT for 1 hour. After a final washing step, 100 µl of SIGMAFAST o-phenylenediamine dihydrochloride substrate solution were added to each well at RT for 10 minutes. The reaction was stopped by addition of 50 µl/well of 3M HCl. Optical density at 490 nm was measured using a Synergy 4 plate reader. Purified human plasma IgA (EMD Millipore, #401098) was diluted (starting at 500 ng/ml and serially diluted two-fold) and was used as standard. A five-parameter logistic fit was conducted on the standard curve and IgA concentrations within saliva samples were calculated.

Statistical analysis

Differences between antibody titers between two groups were analyzed with a ratio paired t test. All statistical analyses, AUC calculation, and five-parameter logistic fit of standard curves were conducted with Graphpad Prism version 10.

References

1. Simon V, Kota V, Bloomquist RF, Hanley HB, Forgacs D, Pahwa S, Pallikkuth S, Miller LG, Schaenman J, Yeaman MR, Manthei D, Wolf J, Gaur AH, Estepp JH, Srivastava K, Carreño JM, Cuevas F, Ellebedy AH, Gordon A, Valdez R, Cobey S, Reed EF, Kolhe R, Thomas PG, Schultz-Cherry S, Ross TM, Krammer F, PARIS/SPARTA Study Group. 2022. PARIS and SPARTA: Finding the Achilles' Heel of SARS-CoV-2. mSphere 7:e0017922.
2. Amanat F, Stadlbauer D, Strohmeier S, Nguyen THO, Chromikova V, McMahon M, Jiang K, Arunkumar GA, Jurczyszak D, Polanco J, Bermudez-Gonzalez M, Kleiner G, Aydillo T, Miorin L, Fierer DS, Lugo LA, Kojic EM, Stoever J, Liu STH, Cunningham-Rundles C, Felgner PL, Moran T, García-Sastre A, Caplivski D, Cheng AC, Kedzierska K, Vapalahti O, Hepojoki JM, Simon V, Krammer F. 2020. A serological assay to detect SARS-CoV-2 seroconversion in humans. Nat Med.
3. Stadlbauer D, Amanat F, Chromikova V, Jiang K, Strohmeier S, Arunkumar GA, Tan J, Bhavsar D, Capuano C, Kirkpatrick E, Meade P, Brito RN, Teo C, McMahon M, Simon V, Krammer F. 2020. SARS-CoV-2 Seroconversion in Humans: A Detailed Protocol for a Serological Assay, Antigen Production, and Test Setup. Curr Protoc Microbiol 57:e100.
4. Hsieh CL, Goldsmith JA, Schaub JM, DiVenere AM, Kuo HC, Javanmardi K, Le KC, Wrapp D, Lee AG, Liu Y, Chou CW, Byrne PO, Hjorth CK, Johnson NV, Ludes-Meyers J, Nguyen AW, Park J, Wang N, Amengor D, Lavinder JJ, Ippolito GC, Maynard JA, Finkelstein IJ, McLellan JS. 2020. Structure-based design of prefusion-stabilized SARS-CoV-2 spikes. Science 369:1501-1505.
